# Monosodium Glutamate Induces Changes in Hepatic and Renal Metabolic Profiles and Gut Microbiome of Wistar Rats

**DOI:** 10.3390/nu13061865

**Published:** 2021-05-30

**Authors:** Kanokwan Nahok, Jutarop Phetcharaburanin, Jia V. Li, Atit Silsirivanit, Raynoo Thanan, Piyanard Boonnate, Jarus Joonhuathon, Amod Sharma, Sirirat Anutrakulchai, Carlo Selmi, Ubon Cha’on

**Affiliations:** 1 Department of Biochemistry, Faculty of Medicine, Khon Kaen University, Khon Kaen 40002, Thailand; Kanokwan.nahok@gmail.com (K.N.); jutarop@kku.ac.th (J.P.); atitsil@kku.ac.th (A.S.); raynoo@kku.ac.th (R.T.); piyanard_nick@hotmail.com (P.B.); 2 Chronic Kidney Disease Prevention in the Northeast Thailand (CKDNET), Khon Kaen University, Khon Kaen 40002, Thailand; amodssharma@gmail.com (A.S.); sirirt_a@kku.ac.th (S.A.); 3 Department of Metabolism, Digestive Disease and Reproduction, Faculty of Medicine, Imperial College London, South Kensington, London SW7 2AZ, UK; jia.li@imperial.ac.uk; 4 Northeast Laboratory Animal Center, Khon Kaen University, Khon Kaen 40002, Thailand; Jarus@kku.ac.th; 5 Department of Internal Medicine, Faculty of Medicine, Khon Kaen University, Khon Kaen 40002, Thailand; 6 Rheumatology and Clinical Immunology, Humanitas Clinical and Research Center IRCCS, Rozzano, 20089 Milan, Italy; 7 Department of Clinical Biosciences, Humanitas University, Pieve Emanuele, 20090 Milan, Italy

**Keywords:** monosodium glutamate, gut microbiota, metabolic pathway, metabolomics, microbiome, trimethylamine

## Abstract

The short- and long-term consumption of monosodium glutamate (MSG) increases urinary pH but the effects on the metabolic pathways in the liver, kidney and the gut microbiota remain unknown. To address this issue, we investigated adult male Wistar rats allocated to receive drinking water with or without 1 g% MSG for 2 weeks (*n* = 10, each). We performed a Nuclear Magnetic Resonance (NMR) spectroscopy-based metabolomic study of the jejunum, liver, and kidneys, while faecal samples were collected for bacterial DNA extraction to investigate the gut microbiota using 16S rRNA gene sequencing. We observed significant changes in the liver of MSG-treated rats compared to controls in the levels of glucose, pyridoxine, leucine, isoleucine, valine, alanine, kynurenate, and nicotinamide. Among kidney metabolites, the level of trimethylamine (TMA) was increased, and pyridoxine was decreased after MSG-treatment. Sequencing of the 16S rRNA gene revealed that MSG-treated rats had increased Firmicutes, the gut bacteria associated with TMA metabolism, along with decreased *Bifidobacterium* species. Our data support the impact of MSG consumption on liver and kidney metabolism. Based on the gut microbiome changes, we speculate that TMA and its metabolites such as trimethylamine-*N*-oxide (TMAO) may be mediators of the effects of MSG on the kidney health.

## 1. Introduction

Monosodium glutamate (MSG) is commonly added to foods to increase palatability, especially in Asian cuisine and industrially processed foods [1]. MSG is considered a safe ingredient for human consumption regardless of the amount by the Food and Drug Administration (FDA) [2] despite recent data showing that the average daily MSG intake is 3–4 g and that every additional gram of MSG increases the risk of developing metabolic syndrome [3]. Elevated amounts of dietary MSG are associated with overweight [4,5] and hypertension [6], but the results are inconsistent [7,8].

Attempts have been made to reveal the effects of MSG on metabolic organs of animals by either parenteral [9,10] or oral intake [11,12]. In one of such efforts to investigate the effects of MSG in rat kidneys, the results showed that both short- [13] and long-term [11] MSG consumption caused alkaline urine in rats, although the mechanism of urine alkalinization is not established. Metabolomics is a commonly used approach to define the changes in metabolites from various conditions [14,15]. The short-term MSG consumption caused specific changes in urinary metabolites including dimethylamine (DMA) and methylamine (MA), which are gut-derived metabolites from trimethylamine (TMA). TMA is a volatile short-chain aliphatic amine that provides the characteristic fish odour. In fact, the gut bacteria produce TMA from dietary fish or it can be generated from other nutrients including choline and carnitine, which are abundant in eggs and red meat [16]. The major part of TMA is enzymatically converted to the odourless trimethylamine-*N*-oxide (TMAO), and high TMAO plasma levels are associated with cardiovascular disease (CVD) [17], chronic kidney disease (CKD) [18] and diabetes [19].

We herein took advantage of metabolomics tools to investigate the effects of short-term MSG consumption on the metabolome of plasma, liver, kidney and gut, and analysed the correlation between the metabolomic results and the changes of the gut microbiota. Our data may provide new mechanistic insights into the effects of dietary MSG while also demonstrating biomarkers of MSG-induced organ damage.

## 2. Materials and Methods

### 2.1. Animals

Twenty 6-week-old male Wistar rats (weight approximately 200 g) were obtained from the National Laboratory Animal Center (Mahidol University, Salaya, Bangkok, Thailand), acclimatized in individual stainless-steel metabolic cages for 2 weeks, and then maintained under a standard condition of temperature (23 ± 2 °C), humidity (30–60%) and brightness (350–400 Lux) of 12 h dark/12 h light cycle. Rats were provided with a commercial pellet diet No. CP 082 (Perfect Companion Group, Bangkok, Thailand) during the study. All experiments were performed following the guidelines of the Northeast Laboratory Animal Center (NELAC), Khon Kaen University, Thailand. Moreover, the study was approved by the Animal Ethics Committee of Khon Kaen University, Thailand (AEKKU-NELAC 5/2558).

### 2.2. Reagents

Pure food-grade (99%) MSG (Ajinomoto, Tokyo, Japan) was used to feed the animals. Commercial grade methanol and chloroform were purchased from RCI LABSCAN LIMITED (Bangkok, Thailand) for tissue extraction, LC-MS-grade water was purchased from Merck (Darmstadt, Germany), potassium dihydrogen phosphate (KH_2_PO_4_) and deuterium oxide (D_2_O) were purchased from Merck (Darmstadt, Switzerland), sodium azide (NaN_3_) was produced by the European Chemicals Agency (ECHA, Helsinki, Finland), sodium trimethylsilyl-[2,2,3,3-^2^H_4_]-propionate (TSP), an internal standard for NMR analysis, was obtained from Santa Cruz Biotechnology (Santa Cruz CA, USA). For mass spectrometry, commercial-grade isopropanol (C_3_H_8_O) and formic acid (CH_2_O_2_) were purchased from RCI LABSCAN LIMITED (Bangkok, Thailand).

### 2.3. Experimental Design

Rats were randomly assigned to receive drinking water either with (n = 10) or without (n = 10) 1 g% MSG for 2 weeks. Reverse osmosis (RO) water was used (chlorine concentration of 3–4 ppm) for the animal study when all the rats had free access to food. Daily intake of water (ml) and food (g) were recorded, as well as the weekly body weight (g). The 24 h urinary excretion (ml/rat/day) was recorded throughout the study period. Faeces and tail blood samples (100 µL of plasma) were collected at 2 weeks prior to the sacrifice of animals using carbon dioxide (CO_2_) after a 12-h fasting. Tissue samples, i.e., jejunum, liver and kidney, were collected and snap-frozen in liquid nitrogen before being stored at −80 °C until used for analysis.

### 2.4. Sample Preparation and Analysis

Each tissue (100 mg wet mass) was used for metabolite extraction according to the previously published protocols [20]. Before NMR acquisition, the polar phase of tissue extracts was re-suspended in an NMR buffer of 580 µL (100 mM sodium phosphate buffer, pH 7.4, in D_2_O, containing 0.1 mM TSP and 0.2% NaN_3_), vortexed briefly, and centrifuged at 12,000× *g* for 5 min at 4 °C. Subsequently, 550 µL of the mixture was transferred to an NMR glass tube (Duran Group, Mainz, Germany) with an outer diameter of 5 mm prior to NMR analysis. Approximately 250 mg of faeces was mixed with 500 µL HPLC-grade water (Merck, Darmstadt, Germany) and homogenized using a vortex mixer with the speed 2500 rpm for 15 min at room temperature. The faecal suspension was then centrifuged at 12,000× *g* for 15 min at 4 °C and 540 µL of supernatant was transferred into clean 1.5 mL microtubes, and 60 µL of NMR buffer (1.5 M KH_2_PO_4_ buffer, pH 7.4, in D_2_O, containing 2 mM TSP and 1% NaN_3_) was added. Next, the tubes were vortexed briefly, centrifuged at 12,000× *g* for 10 min, and finally, 580 µL of the mixture was transferred to a 5 mm outer diameter NMR glass tube for analysis.

Tissue extracts and faecal samples were analysed using a 400 MHz NMR spectrometer (Bruker Biospin, Rheinstetten, USA) at a temperature of 298.15 K. The spectra were referenced to the TSP peak (δ^1^H 0.00), phased and baseline corrected using MATLAB (Mathworks, Natrick, MA USA). The signal of TSP peak (δ^1^H −1.000–0.005) from all tissues and TSP peak (δ^1^H −1.20–0.157) from faeces were removed. In addition, the water peak was removed from the jejunum (δ^1^H 4.50 and 5.20), liver (δ^1^H 4.68 and 5.00), kidney (δ^1^H 4.31 and 5.74) and faeces (δ^1^H 4.18 and 5.23). Moreover, raw spectra were subjected to the peak alignment and normalization [21]. The spectral data of all samples were analysed using unsupervised principal component analysis (PCA) and supervised orthogonal signal correction-projection to latent structures-discriminant analysis (O-PLS-DA). The data were mean-centred and scaled with unit variance (UV). The O-PLS-DA models are evaluated by the R^2^X, R^2^Y and Q^2^Y values, representing the fitness, fraction of variances of Y matrix, and forecasting ability of the model, respectively [22]. To avoid over-fitting, 7-fold cross-validation was performed for 500 repeats. Permutation *p*-value was used to indicate the model validity. Significant variables of each valid model were selected through O-PLS-DA correlation coefficients with Benjamini–Hochberg false-discovery rate correction (*p* < 0.05). Statistical total correlation spectroscopy (STOCSY) [23], in-house chemical shift databases and the Human Metabolome Database (HMDB version 4, USA) [24] were used for metabolite identification.

Pathway enrichment analysis was also conducted using MetaboAnalyst (http://www.metaboanalyst.ca/ (accessed on 14 July 2020)) [25]. The metabolic pathway illustration was generated by Cytoscape [26]. Changes in metabolites were identified by STOCSY and univariate analysis was performed by investigating the relative concentration of significantly differential metabolites, calculating the integration of spectra peaks. According to the *p*-value calculated by the Student’s *t*-test using GraphPad Prism 7 (Ver. 7, GraphPad Software, Inc., La Jolla, CA, USA).

### 2.5. Plasma Sample Preparation for UHPLC-ESI-QTOF-MS Analysis

Plasma (50 µL) was mixed with 150 µL of isopropanol (IPA), followed by 24-h incubation at 20 °C to precipitate the protein. All samples were centrifuged twice at 13,000× *g* at 4 °C for 10 min. A total of 50 µL was taken from each sample and pooled in a 1.5 mL microcentrifuge tube for constructing a quality control (QC) sample and 120 µL of each sample mixture was transferred to an HPLC glass insert.

### 2.6. UHPLC-ESI-QTOF-MS Analysis

UHPLC-ESI-QTOF-MS analysis was employed at Khon Kaen University International Phenome Laboratory (KKUIPL). The aqueous phase extracts of samples were analysed on a reverse-phase platform. The separation part was performed using a UHPLC system (Bruker, Darmstadt, Germany) when the Bruker intensity solo HPLC C18 2.1 × 100 mm, 2 µm column (Bruker, Darmstadt, Germany) was used. The column temperature was set at 55 °C and the autosampler temperature was set at 4 °C. Mobile phase A was 100% HPLC grade water with 0.1% formic acid (FA), and mobile phase B was 100% methanol with 0.1% FA. The flow rate was set at 0.4 mL/min and the elution gradient was set as—99.9% A (0.0–2.0 min, 0.25 mL/min), 99.9–75% A (2.0–10.0 min, 0.4 mL/min), 20% A (10.0–12.0 min, 0.4 mL/min), 10% A (12.0–21.0 min, 0.4 mL/min), 0.1% A (21.0–23.0 min, 0.4 mL/min), 99.9% A (24.0–26.0 min, 0.4 mL/min). A sample injection volume of 4 µL was applied for both positive and negative ionization polarity modes.

Mass spectrometry analyses were performed using a compact ESI-Q-TOF system (Bruker, Darmstadt, Germany). Sodium formate (HCOONa) containing 2 mM sodium hydroxide, 0.1% FA and 50% IPA was directly injected as an external calibrant with flow rate 0.5 µL/min. The condition in positive ionization polarity mode—mass range 50–1300 m/z, cone voltage 31 V, capillary voltage 4500 V, source temperature 220 °C, desolvation temperature 220 °C, desolvation gas flow 8 L/min. The conditions of negative ionization polarity mode—m/z range: 50–900 m/z, cone voltage 31 V, capillary voltage 4500 V, source temperature 220 °C, desolvation temperature 220 °C, desolvation gas flow 8 L/min.

### 2.7. Gut Microbiome Analysis

DNA was extracted using the QiAamp^®^ PowerFecal^®^ Pro DNA kit (Qiagen, Hilden, Germany). Extracted DNA was measured at OD 260/280 using a Nanodrop2000c spectrophotometer (Thermo scientific, Waltham, MA, USA). All of the extracted DNA were preserved at −20 °C until analyses. The V3-V4 region of the 16S ribosomal RNA (rRNA) gene for each sample was amplified using the universal forward primer V3 (5′-CCTACGGGNGGCWGCAG-3′) and the reverse primer V4 (5′-GACTACHVGGGTATCTAATCC-3′). PCR reaction consisted of 2x KAPA HiFi HotStart ReadyMix, 12.5 ng DNA template, and 5 µM of each primer, with initial denaturation at 95 °C for 3 min followed by 25 cycles of denaturation at 95 °C for 30 s, annealing at 55 °C for 30 s, extension at 72 °C for 30 s, and final extension at 72 °C for 10 min. An Agilent DNA 1000 Chip and Agilent 2100 Bioanalyzer (Agilent Technologies, Palo Alto, CA, USA) were combined to quantify the amplified product. PCR amplicons were purified using AMPure XP beads to purify. The partial 16S rRNA gene was sequenced using Illumina MiSeq platform (Illumina Inc., San Diego, CA, USA).

The 16S rRNA sequencing library was prepared using genomic DNA (gDNA), and matching paired-end sequences were merged using FLASH (http://ccb.jhu.edu/software/FLASH/ (accessed on 29 June 2020)) [27]. Quality filtering removed sequences containing low-quality reads and was performed using CD-HIT-OTU (http://weizhong-lab.ucsd.edu/cd-hit-otu (accessed on 29 June 2020)) [28] and the rDnaTools package. OTUs (Operational taxonomic units) were classified with 97% threshold identity using UCLUST algorithm based on 100% similarity. Sequences sharing ≥97% similarity were assigned to the same OTU. Representative OTU sequences were aligned and were taxonomically classified using Ribosomal Database Project (RDP) and were compared with the reference NCBI databases. The classification is based on the Green genes database (http://greengenes.lbl.gov/ (accessed on 29 June 2020)). The output of a classification of reads at several taxonomic levels—kingdom, phylum, class, order, family, genus, and species using Quantitative Insights Into Microbial Ecology (QIIME) [29].

### 2.8. Statistical Analysis

The statistical analysis of the food and water intake, urine volume output and body weight were reported as mean ±SD per group of animals and the differences between groups were compared for statistical significance by Student’s *t*-test with *p*-value < 0.05 considered as statistically significant.

## 3. Results

### 3.1. IMPACT of MSG on Food and Water Intake, Body Weight and Urine Volume Output

**Figure 1:**
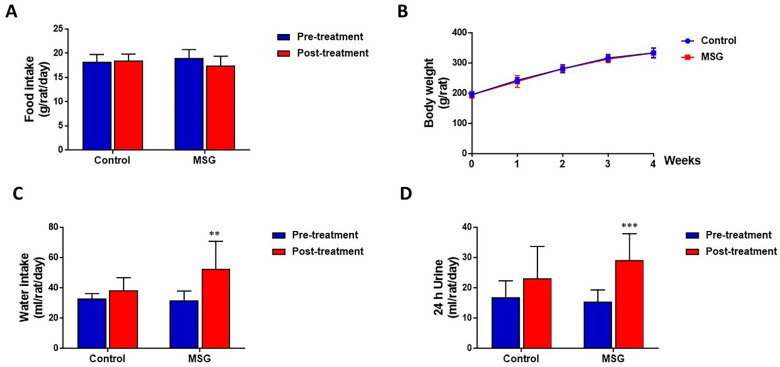
(**A**) Food intake (**B**) Body weight (**C**) Water intake and (**D**) Urine output of male Wistar rats supplemented with 1 g% MSG and control animals (n = 10 per group) at baseline and at 2 weeks. Data are shown as mean ± SD and *p*-values calculated by Student’s *t*-test, ** *p* < 0.01, *** *p* < 0.001).

MSG-treated and control rats had a similar amount of food intake (17.44 ± 1.94 and 18.46 ± 1.37 g/rat/day, respectively) (Figure 1A) and body weight (333.58 ± 17.23 and 333.80 ± 15.50 g/rat, respectively) (Figure 1B). However, water intake was significantly higher in MSG-treated rats (52.38 ± 18.36 mL/day) compared to controls (38.38 ± 8.39 mL/day) (Figure 1C). In both MSG-treated and control groups, urine output tended to increase with time, although a significant increase was seen only in MSG-treated rats (15.42 ± 3.92 mL/rat/day at pre-treatment and 29.09 ± 8.78 mL/rat/day post-treatment). Also, although statistically not significant, urine output of MSG-treated rats (29.09 ± 8.78 mL/rat/day) was apparently higher than that of controls (23.15 ± 10.55 mL/rat/day) (Figure 1D).

### 3.2. Metabolic Changes in Metabolically Important Organs

**Figure 2:**
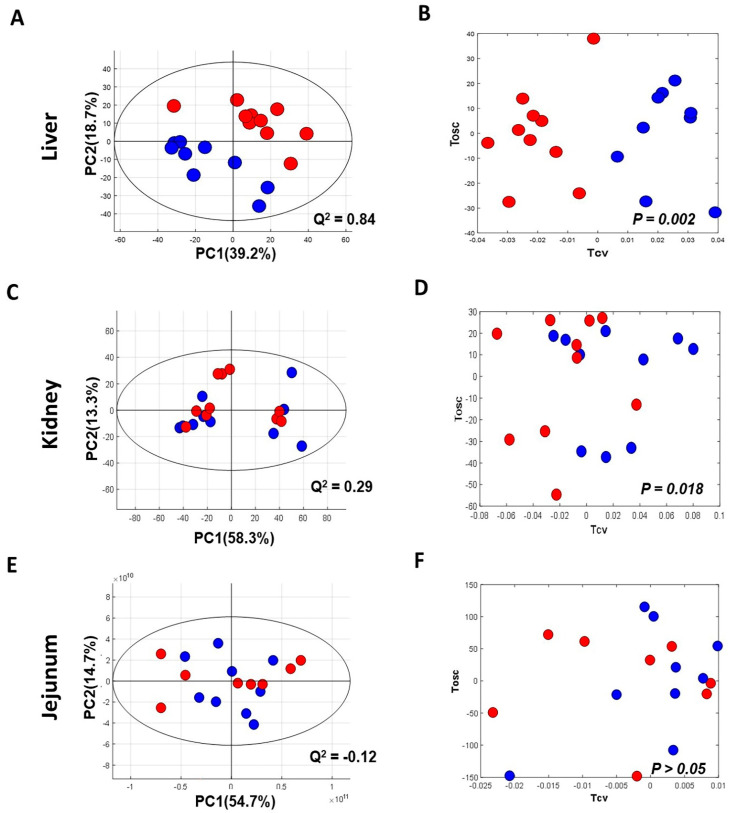
Principal component analysis (left panel) and O-PLS-DA score plot (right panel) of tissues of MSG-treated (Red) and control groups (Blue). (**A**,**B**) Liver; (**C**,**D**) kidney; and (**E**,**F**) jejunum.% PC represents variation explained by each principal component and Q^2^ represents predictive ability of the model.

**Figure 3:**
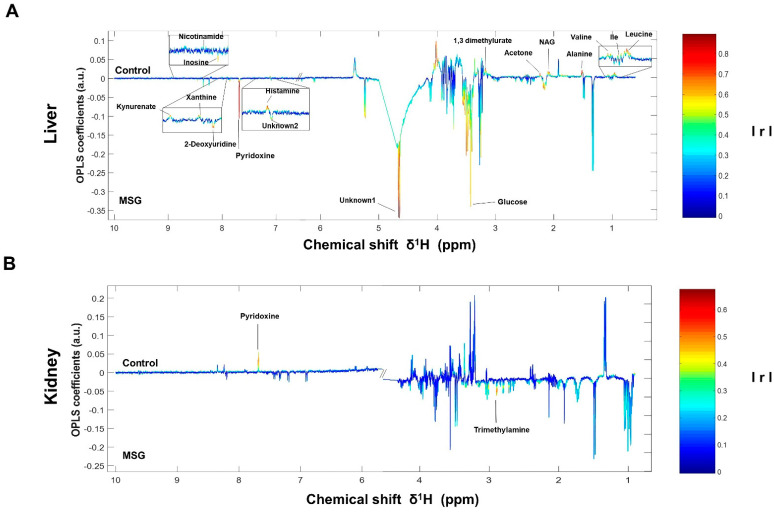
OPLS-corresponding coefficient loading plots of liver (**A**) and kidney (**B**). Positive and negative signals denote significant metabolites in MSG-treated rats (downwards pointing) and control groups (upwards pointing) with intensity indicated by colour (red is highly correlated and blue is weakly correlated) and also correlation per metabolite. Abbreviations: Ile, isoleucine; NAG, *N*-acetylglycoprotein; IrI, correlation coefficients.

The NMR spectra of the collected tissue samples were analysed after two weeks of MSG treatment. PCA of NMR spectral data was initially carried out to observe any obvious clustering and outliers (Figure 2). The clear clustering and complete separation were observed in hepatic metabolite profiles between the control and MSG-treated group with a permutation *p*-value of 0.002, R^2^X of 58%, Q^2^Y of 0.84, and R^2^Y of 0.93 illustrated in PCA and O-PLS-DA score plots (Figure 2A, B). A representative OPLS-corresponding coefficient loading plot with metabolite assignment is presented in Figure 3 with significant liver models indicated in Figure 3A; all significant changes in metabolites are summarized in Table 1. In the liver, higher levels of six metabolites including glucose, pyridoxine, 2-deoxyuridine, inosine, unknown 1 and 2 were found in the MSG-treated group, whereas eleven metabolites, namely leucine, isoleucine, valine, alanine, *N*-acetylglycoprotein, acetone, 1,3 dimethylurate, histamine, xanthine, kynurenate and nicotinamide, were significantly higher in the control group. The PCA score plot based on the renal metabolites (Figure 2C) indicated no clear clustering between two groups, and the O-PLS-DA model was statistically significant with a permutation *p*-value of 0.018, R^2^X of 56%, Q^2^Y of 0.29, and R^2^Y of 0.74 (Figure 2D). Two altered metabolites were found in kidney tissues with significantly higher trimethylamine level and significantly lower pyridoxine in the MSG-treated group compared to controls (Figure 3B). In contrast, the results of jejunum (Figure 2E, F), faeces and plasma (Figure 2 and Figure S1) revealed no clustering between the treatment and control groups illustrated in PCA and O-PLS-DA scores plots.

The correlation pathways of the significant metabolites in the liver and kidney tissues were investigated using KEGG IDs and generated by Cytoscape software. The metabolites were found to link the metabolic network. When these results were combined to draw a metabolic pathway map in order to illustrate a more intuitive correlation, the metabolites were revealed to be mainly associated with metabolic pathways, such as hepatic gluconeogenesis, branched-chain amino acids, vitamin B6, and trimethylamine metabolisms (Figure S3).

### 3.3. MSG Consumption Alters Gut Microbiome

**Figure 4:**
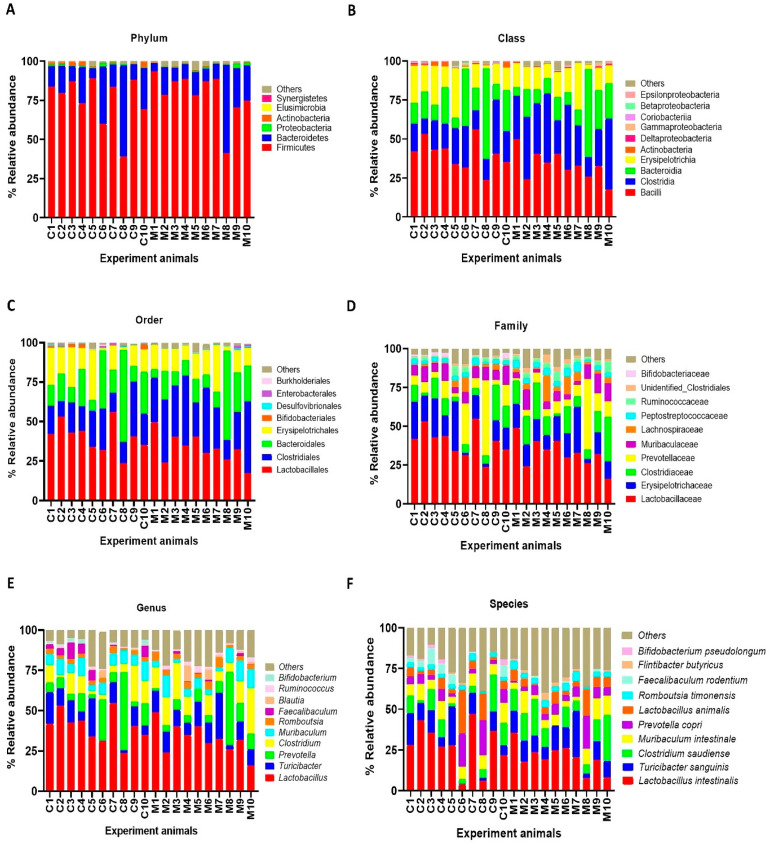
Faecal microbial composition at the different taxonomic levels in MSG-treated (M1–M10) and control (C1–C10) rats. (**A** = phylum, **B** = class, **C** = order, **D** = family, **E** = genus and **F** = species). Others indicate other bacteria and unidentified bacteria <5%.

**Figure 5:**
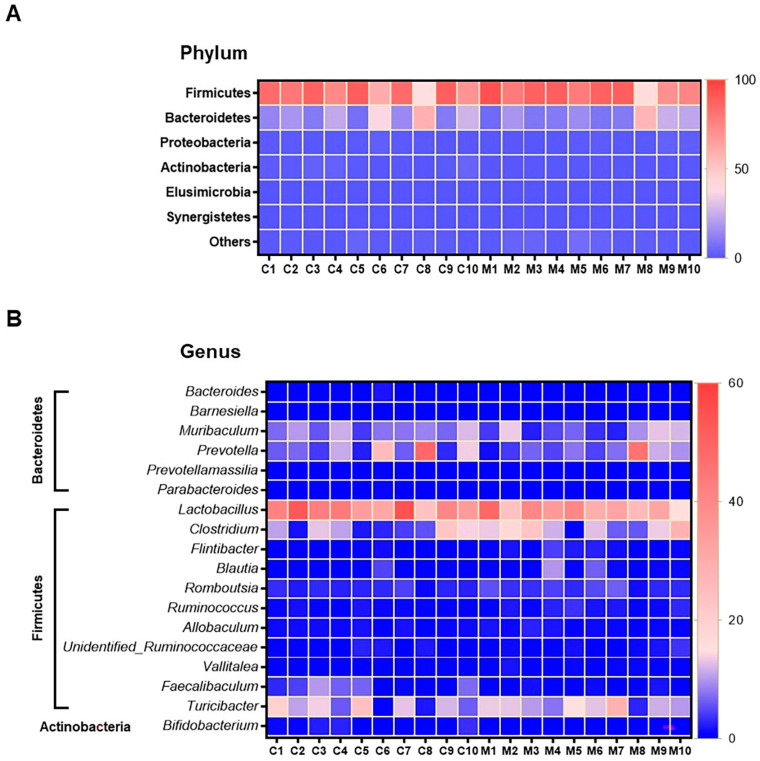
Heatmap of relative operational taxonomic unit (OTU) abundance at the phylum level (**A**), and genus level (**B**) from faeces samples of control (*n* = 10) and the MSG group (*n* = 10). The colour key corresponds to percent relative abundance of the gut microbiota in OTU at each expression level.

The results of faecal bacterial composition analysis indicated seven phyla, 15 classes, 19 orders, 46 families, 127 genera and 220 species from all animal groups. The top 7–10 species were selected and percentage relative abundance of bacterial communities at the phylum, class, order, family, genus and species levels was generated (Figure 4). At the phylum level, the top four relatively abundant phyla were in the following order—Firmicutes > Bacteroidetes > Proteobacteria > Actinobacteria in all animals (Figure 4A). The abundance of Actinobacteria was significantly lower in MSG-treated rats (0.30%) than in control rats (1.32%) (Figure 5A).

At the class level, the most abundant microbiota were Bacilli > Clostridia > Bacteroidia > Erysipelotrichia in all animals (Figure 4B). The MSG-treated group had significantly higher Clostridia (31.59%) than the control group (19.25%). However, the MSG-treated rats had significantly lower Actinobacteria (0.15%) than control rats (1.09%) (Figure 4B). When interpreted at the order level, Lactobacillales, Clostridiales, Bacteroidales, Erysipelotrichales were abundant in all animals (Figure 4C). Clostridiales were significantly higher in MSG-treated rats (31.59%) than in control rats (19.25%), while Bifidobacteriales were significantly lower in the MSG-treated rats (0.06%) compared to control rats (1.06%).

The four most abundant families commonly observed in all experimental animals were in the following order—Lactobacillaceae > Erysipelotrichaceae > Clostridiaceae > Prevotellaceae (Figure 4D). At the family level, Bifidobacteriaceae was significantly lower, but unidentified Clostridiales was significantly higher in MSG-treated rats compared to control rats. The relative abundance of top four genera observed in all the animals were in the following order—*Lactobacillus > Turicibacter > Prevotella < Clostridium* (Figure 4E). A heatmap of the relative abundances at the genus level showed lower abundance of *Lactobacillus* and higher abundance of *Clostridium* in MSG-treated rats (Figure 5B). Moreover, the lower abundance of *Bifidobacterium* was also observed in MSG-treated rats (0.06%) compared to control rats (1.06%).

At the species level, the top four gut bacterial species common in all rats were *Lactobacillus intestinalis,* *Turicibacter sanguinis, Clostridium saudiense,* and *Muribaculum intestinale* in order (Figure 4F). In the MSG-treated group, *Flintibacter butyricus* was significantly more abundant than in the control group, whereas *Faecalibaculum rodentium* and *Bifidobacterium pseudolongum* were significantly less abundant.

## 4. Discussion

Several lines of evidence have consistently linked MSG consumption to adverse effects on human health, such as higher incidence of metabolic syndrome [3], overweight [4,5] and arterial hypertension [6]. Nonetheless, data have been conflicting in some cases and the underlying mechanisms remain unclear. To tackle this issue, we explored the tissue metabolic alterations and the gut microbial changes induced by MSG consumption.

**Figure 6:**
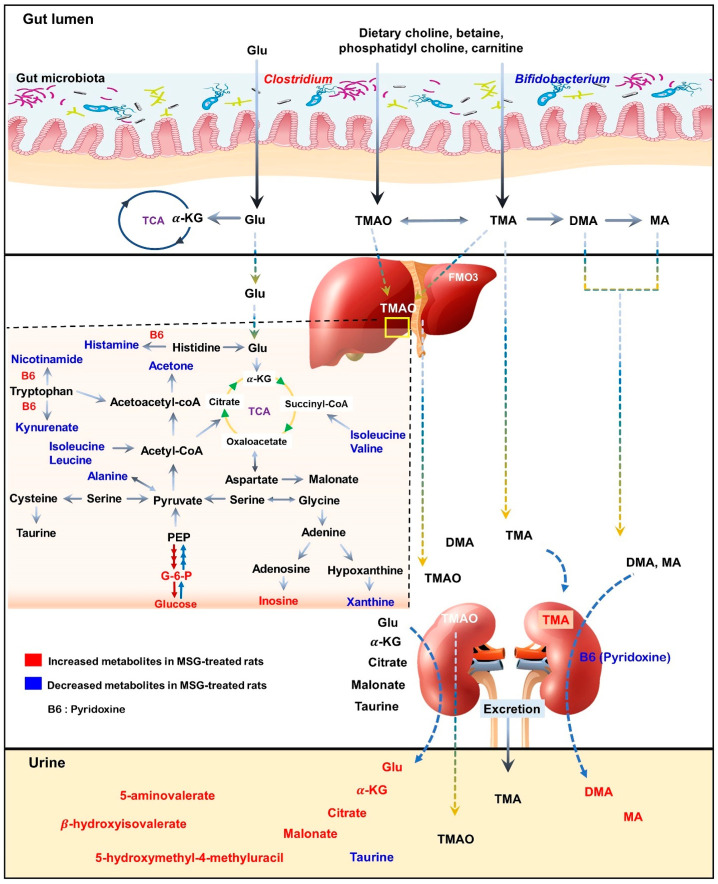
Schematic illustration of changes in metabolic pathways as observed from tissues and biofluid metabolites analysis of Wistar rats. Red indicates higher relative concentration, whereas blue indicates lower relative concentration of metabolites in control rats compared with MSG-treated rats.

As illustrated in the schematic representation in Figure 6, MSG consumption altered the metabolites associated with hepatic gluconeogenesis, branched-chain amino acid (BCAA) metabolism, vitamin B6 and trimethylamine metabolism. Moreover, the short-term MSG consumption suppressed the relative abundance of *Bifidobacterium*, which is considered a beneficial bacteria, and *Clostridium* related to TMA and TMAO production [30].

In the liver, metabolites such as glucose, pyridoxine (B6), 2-deoxyuridine, inosine, unknown 1 and 2 were significantly higher in MSG-treated animals than in controls, while eleven metabolites (i.e., leucine, isoleucine, valine, alanine, *N*-acetylglycoprotein, acetone, 1,3-dimethylurate, histamine, xanthine, kynurenate, and nicotinamide) were significantly lower. First, higher levels of glucose may indicate an increase of hepatic gluconeogenesis associated with MSG, which is in agreement with previously reported high plasma glucose in newborn pigs receiving MSG supplementation (1 g/kg body weight) [31]. Second, the reduced levels of alanine and three BCAAs, including valine, leucine and isoleucine, may correlate with amino acid catabolism and energy metabolism in which their carbon skeletons can be used as precursors for gluconeogenesis (alanine, valine and isoleucine as glucogenic amino acids) and energy production via the tricarboxylic acid (TCA) cycle. In agreement with the previous report, we found that the degradation products of leucine and lysine, beta-hydroxyisovalerate and 5-aminovalerate, respectively, were significantly higher in the urine of MSG-treated rats [13]. Third, elevated pyridoxine in the liver and decreased pyridoxine in the kidney of MSG-treated rats suggested alteration of vitamin B6 metabolism. We also found decreased levels of histamine, kynurenate, and nicotinamide in the liver of MSG-treated rats, which are the products of B6-dependent enzymes in histidine and tryptophan metabolism. The connection between MSG intake and metabolism of vitamin B6, histidine and tryptophan need to be further investigated. Fourth, MSG-treated rats had higher levels of trimethylamine (TMA) in the kidney. TMA is synthesized from dietary constituents, including choline, L-carnitine and betaine by the action of microbial enzymes [16]. TMA is absorbed largely in a passive way into the portal circulation, and mainly oxidized to trimethylamine-*N*-oxide (TMAO) by hepatic flavin-containing monooxygenases (FMOs). A minor fraction of TMA is oxidized to dimethylamine (DMA) and methylamine (MA), and finally excreted into urine [32,33]. Higher levels of DMA and MA in the urine of MSG-treated rats were reported previously [13]. Several studies have demonstrated that the elevated TMA precursors, choline, or its metabolites, TMAO, can lead to progressive renal tubulointerstitial fibrosis, cardiovascular disease, and chronic kidney disease [34,35,36]. Therefore, the elevated level of TMA in the kidney may constitute the link between MSG intake and renal damage.

Moving from the observed changes in TMAs, we investigated the gut microbiota of MSG-treated rats and demonstrated the alteration of the two major phyla, Firmicutes and Bacteroidetes. The higher abundance of Firmicutes, but lower abundance of Bacteroidetes, was observed in MSG-treated rats. At the genus level, MSG-treated rats had higher abundance of *Clostridium,* and lower abundance of *Lactobacillus* and *Bifidobacterium*. The genus *Clostridium* comprises a group of microorganisms belonging to the phylum Firmicutes. Some *Clostridium* spp. are associated with TMA metabolism by producing enzymes to convert choline or dietary constituents to TMA [33,37,38]. The increased TMA-producing gut bacteria, i.e., *Clostridium* spp., supports the elevation of TMA metabolites in the kidney and urine of MSG-treated rats. Moreover, MSG consumption reduced the *Bifidobacterium* population, a probiotic bacterium which plays important roles in gut homeostasis and health [39]. Simple and digestible carbohydrates such as lactose and sucrose are metabolized in the upper gastrointestinal (GI) tract by the host and bacteria such as lactobacilli. However, non-digestible carbohydrates, i.e., dietary fibre, are metabolized in the lower GI tract by the members of gut microbiota, including *Bifidobacterium*. A diet with high contents of saturated fats and simple sugars but depleted of dietary fibre such as a western-style diet may contribute to a lower number of beneficial bacteria [40]. The decrease of *Bifidobacterium,* a good bacterium, has been reported in chronic inflammatory diseases such as obesity [41], hepatitis B [42], and diabetes [43,44]. The effect of MSG consumption on the gut microbiota in humans was reported earlier with no significant changes in microbial composition compared to the baseline [45]. The low impact of MSG on the gut microbiota in this study may be due to the low dose of MSG supplementation (2 g/day), since the average daily MSG intake in our observation is 4 g/day [3]. We found that every 1 g of daily MSG intake increased the risk of having metabolic syndrome. Although the mechanism of MSG consumption leading to metabolic diseases is not established, the decrease of *Bifidobacterium* in MSG-treated animals may be a clue. Coincidently, the decreased *Bifidobacterium* in MSG-treated rats found in present study is similar to that of rats with vitamin B6 deficiency [46]. The intensive study of how MSG reduces good bacteria and alters vitamin B6 status needs further investigation.

We showed that MSG consumption induced hepatic and renal metabolic changes involved in gluconeogenesis and branched-chain amino acid, vitamin B6 and TMA metabolism, accompanied by gut microbiota compositional shifts. These observations suggest that the altered metabolic pathways may be associated with adverse effects of long term of MSG consumption, especially on the liver and kidney.

## Figures and Tables

**Table 1:** Relative changes in liver and kidney metabolites of control and MSG-treated rats, using the ^1^H NMR profiles.

Metabolites	Chemical SHIFT (Multiplicity)	MSG Induced Metabolic Changes Compared to Control	
		(−) Control vs. (+) MSG R2X = 58%, Q2Y = 0.84, *p* = 0.002 (liver)	(−) Control vs. (+) MSG R2X = 56%, Q2Y = 0.29, *p* = 0.018 (kidney)
Leucine	**0.9644** (**t**), 1.735 (t), 3.787 (m)	−0.7823	-
Isoleucine	0.9644 (t), **1.006** (**d**), 1.476 (d), 3.833 (d)	−0.8539	-
Valine	0.9644 (t), 1.006 (d), **1.039** (**d**), 1.476 (d)	−0.7768	-
Alanine	**1.494** (**d**), 4.106 (q)	−0.6109	-
*N*-Acetylglycoprotein	**2.086** (**s**)	−0.8440	-
Acetone	**2.237** (**s**)	−0.8654	-
Trimethylamine	**2.89** (**s**)	-	0.6579
1,3-dimethylurate	**3.306** (**s**), 3.359 (s)	−0.7834	-
Glucose	3.27 (t), **3.426** (**m**), 4.642 (d), 5.234 (d)	0.4626	-
Unknown 1	3.426 (m), 3.521 (m), **4.642** (**d**), 5.233 (d)	0.5254	-
Unknown 2	**7.089** (**s**)	0.7485	-
Histamine	3.095 (m), 3.306 (t), **7.108** (**s**), 7.906 (s)	−0.8240	-
Pyridoxine	**7.684** (**s**)	0.9173	−0.6069
2-Deoxyuridine	3.558 (d), 3.719 (m), 3.833 (m), 5.244 (d), **7.859** (**d**)	0.8117	-
Xanthine	**7.892** (**s**)	−0.7847	-
Kynurenate	**8.198** (**d**)	−0.6075	-
Inosine	6.099 (d), 8.241 (s), **8.348** (**s**)	0.5690	-
Nicotinamide	**8.712** (**d**), 8.945 (s)	−0.6430	-

R^2^X and Q^2^Y show the variance explained and predicted by each model while *p*-values for all models were derived from a permutation test (*n* = 500). (+) Indicates a higher correlation, whereas (−) indicates a lower correlation of urinary metabolites after MSG consumption. The bolded chemical shift per metabolite was used as the STOCSY driver peak and for deriving the correlation and *p*-value. Abbreviations: s, singlet; d, doublet; t, triplet; m, multiple.

## Data Availability

Not applicable.

## Supplementary Materials

The following are available online at https://www.mdpi.com/article/10.3390/nu13061865/s1, Figure S1. Principal component analysis (A) and O-PLS-DA score plot (B) of faeces of MSG treated (Red) and control groups (Blue). % PC represents variation explained by each principal component and Q2 represents predictive ability of the model; Figure S2. Principal component analysis (left panel) and O-PLS-DA score plot (right panel) of plasma after MSG treatment (Red) compared to control group (Blue). (A-B) positive ionization mode and (C-D) negative ionization mode. % PC represents variation explained by each principal component and Q2 represents predictive ability of the model; Figure S3. Pathway analysis of significant metabolites in rat tissues after MSG-treatment. Red frames indicate increased metabolites and green frames indicate decreased metabolites. Abbreviations: G-6-P, glucose-6-phosphate; F-6-P, fructose-6-phosphate; F-1,6-BP, fructose-1,6-bisphosphate; PEP, phosphoenolpyruvate; PEPCK, phosphoenolpyruvate carboxykinase; CPS1, carbamoyl phosphate synthetase1; FMO3, Flavin-containing monooxygenase.

## References

[1] Beyreuther K., Biesalski H.K., Fernstrom J.D., Grimm P., Hammes W.P., Heinemann U., Kempski O., Stehle P., Steinhart H., Walker R. (2006). Consensus meeting: Monosodium glutamate—an update. Eur. J. Clin. Nutr..

[2] Walker R., Lupien J.R. (2000). The Safety Evaluation of Monosodium Glutamate. J. Nutr..

[3] Insawang T., Selmi C., Cha’On U., Pethlert S., Yongvanit P., Areejitranusorn P., Boonsiri P., Khampitak T., Tangrassameeprasert R., Pinitsoontorn C. (2012). Monosodium glutamate (MSG) intake is associated with the prevalence of metabolic syndrome in a rural Thai population. Nutr. Metab..

[4] He K., Du S., Xun P., Sharma S., Wang H., Zhai F., Popkin B. (2011). Consumption of monosodium glutamate in relation to incidence of overweight in Chinese adults: China Health and Nutrition Survey (CHNS). Am. J. Clin. Nutr..

[5] Liancheng Z., Zhao L., Daviglus M.L., Dyer A.R., Van Horn L., Garside D., Zhu L., Guo D., Wu Y., Zhou B. (2008). Association of Monosodium Glutamate Intake With Overweight in Chinese Adults: The INTERMAP Study. Obesity.

[6] Shi Z., Yuan B., Taylor A.W., Dai Y., Pan X., Gill T.K., Wittert G.A. (2011). Monosodium glutamate is related to a higher increase in blood pressure over 5 years: Findings from the Jiangsu Nutrition Study of Chinese adults. J. Hypertens..

[7] Shi Z., Luscombe-Marsh N.D., Wittert G.A., Taylor A.W. (2010). Monosodium glutamate is not associated with obesity or a greater prevalence of weight gain over 5 years: Findings from the Jiangsu Nutrition Study of Chinese adults—response by Shi et al. Br. J. Nutr..

[8] Hien V.T.T., Lam N.T., Khan N.C., Wakita A., Yamamoto S. (2012). Monosodium glutamate is not associated with overweight in Vietnamese adults. Public Heal. Nutr..

[9] Nakanishi Y., Tsuneyama K., Fujimoto M., Salunga T.L., Nomoto K., An J.-L., Takano Y., Iizuka S., Nagata M., Suzuki W. (2008). Monosodium glutamate (MSG): A villain and promoter of liver inflammation and dysplasia. J. Autoimmun..

[10] Nagata M., Suzuki W., Iizuka S., Tabuchi M., Maruyama H., Takeda S., Aburada M., Miyamoto K.-I. (2006). Type 2 Diabetes Mellitus in Obese Mouse Model Induced by Monosodium Glutamate. Exp. Anim..

[11] Sharma A., Prasongwattana V., Cha’On U., Selmi C., Hipkaeo W., Boonnate P., Pethlert S., Titipungul T., Intarawichian P., Waraasawapati S. (2013). Monosodium Glutamate (MSG) Consumption Is Associated with Urolithiasis and Urinary Tract Obstruction in Rats. PLoS ONE.

[12] Boonnate P., Waraasawapati S., Hipkaeo W., Pethlert S., Sharma A., Selmi C., Prasongwattana V., Cha’on U. (2015). Monosodium Glutamate Dietary Consumption Decreases Pancreatic beta-Cell Mass in Adult Wistar Rats. PLoS ONE.

[13] Nahok K., Li J.V., Phetcharaburanin J., Abdul H., Wongkham C., Thanan R., Silsirivanit A., Anutrakulchai S., Selmi C., Cha’On U. (2019). Monosodium Glutamate (MSG) Renders Alkalinizing Properties and Its Urinary Metabolic Markers of MSG Consumption in Rats. Biomolecules.

[14] Putri S.P., Nakayama Y., Matsuda F., Uchikata T., Kobayashi S., Matsubara A., Fukusaki E. (2013). Current metabolomics: Practical applications. J. Biosci. Bioeng..

[15] Nicholson J.K., Wilson I.D. (2003). Opinion: Understanding ‘global’ systems biology: Metabonomics and the continuum of metabolism. Nat. Rev. Drug Discov..

[16] Zeisel S.H., Warrier M. (2017). Trimethylamine N-Oxide, the Microbiome, and Heart and Kidney Disease. Annu. Rev. Nutr..

[17] Wang Z., Klipfell E., Bennett B.J., Koeth R.A., Levison B.S., Dugar B., Feldstein A.E., Britt E.B., Fu X., Chung Y.-M. (2011). Gut flora metabolism of phosphatidylcholine promotes cardiovascular disease. Nat. Cell Biol..

[18] Bain M.A., Faull R., Fornasini G., Milne R.W., Evans A.M. (2006). Accumulation of trimethylamine and trimethylamine-N-oxide in end-stage renal disease patients undergoing haemodialysis. Nephrol. Dial. Transplant..

[19] Dambrova M., Latkovskis G., Kuka J., Strele I., Konrade I., Grinberga S., Hartmane D., Pugovics O., Erglis A., Liepinsh E. (2016). Diabetes is Associated with Higher Trimethylamine N-oxide Plasma Levels. Exp. Clin. Endocrinol. Diabetes.

[20] Beckonert O., Keun H.C., Ebbels T.M.D., Bundy J.G., Holmes E., Lindon J.C., Nicholson J.K. (2007). Metabolic profiling, metabolomic and metabonomic procedures for NMR spectroscopy of urine, plasma, serum and tissue extracts. Nat. Protoc..

[21] Dieterle F., Ross A., Schlotterbeck G., Senn H. (2006). Probabilistic Quotient Normalization as Robust Method to Account for Dilution of Complex Biological Mixtures. Application in 1H NMR Metabonomics. Anal. Chem..

[22] Bartel J., Krumsiek J., Theis F.J. (2013). Statistical Methods for the Analysis of High-Throughput Metabolomics Data. Comput. Struct. Biotechnol. J..

[23] Cloarec O., Dumas M.E., Craig A., Barton R.H., Trygg J., Hudson J., Blancher C., Gauguier D., Lindon J.C., Holmes E. (2005). Statistical total correlation spectroscopy: An exploratory approach for latent biomarker identification from metabolic 1H NMR data sets. Anal. Chem..

[24] Wishart D.S., Feunang Y.D., Marcu A., Guo A.C., Liang K., Vázquez-Fresno R., Sajed T., Johnson D., Allison P., Karu N. (2018). HMDB 4.0: The human metabolome database for 2018. Nucleic Acids Res..

[25] Chong J., Soufan O., Li C., Caraus I., Li S., Bourque G., Wishart D.S., Xia J. (2018). MetaboAnalyst 4.0: Towards more transparent and integrative metabolomics analysis. Nucleic Acids Res..

[26] Kutmon M., Lotia S., Evelo C.T., Pico A.R. (2014). WikiPathways App for Cytoscape: Making biological pathways amenable to network analysis and visualization. F1000Resarch.

[27] Magoč T., Salzberg S.L. (2011). FLASH: Fast Length Adjustment of Short Reads to Improve Genome Assemblies. Bioinformatics.

[28] Li W., Fu L., Niu B., Wu S., Wooley J. (2012). Ultrafast clustering algorithms for metagenomic sequence analysis. Brief Bioinform..

[29] Caporaso J.G., Kuczynski J., Stombaugh J., Bittinger K., Bushman F.D., Costello E.K., Fierer N., Peña A.G., Goodrich J.K., Gordon J.I. (2010). QIIME Allows Analysis of High-Throughput Community Sequencing data. Nat. Methods.

[30] Romano K.A., Vivas E.I., Amador-Noguez D., Rey F.E. (2015). Intestinal Microbiota Composition Modulates Choline Bioavailability from Diet and Accumulation of the Proatherogenic Metabolite Trimethylamine-N-Oxide. mBio.

[31] Stegink L.D., Brummel M.C., Boaz D.P., Filer L.J. (1973). Monosodium Glutamate Metabolism in the Neonatal Pig: Conversion of Administered Glutamate into Other Metabolites in vivo. J. Nutr..

[32] Zeisel S.H., Dacosta K.A., Youssef M., Hensey S. (1989). Conversion of Dietary Choline to Trimethylamine and Dimethylamine in Rats: Dose-Response Relationship. J. Nutr..

[33] Fennema D., Phillips I.R., Shephard E.A. (2016). Trimethylamine and Trimethylamine N-Oxide, a Flavin-Containing Monooxygenase 3 (FMO3)-Mediated Host-Microbiome Metabolic Axis Implicated in Health and Disease. Drug Metab. Dispos..

[34] Tomlinson J.A., Wheeler D.C. (2017). The role of trimethylamine N-oxide as a mediator of cardiovascular complications in chronic kidney disease. Kidney Int..

[35] Xu K.-Y., Xia G.-H., Lu J.-Q., Chen M.-X., Zhen X., Wang S., You C., Nie J., Zhou H.-W., Yin J. (2017). Impaired renal function and dysbiosis of gut microbiota contribute to increased trimethylamine-N-oxide in chronic kidney disease patients. Sci. Rep..

[36] Tang W.W., Wang Z., Kennedy D.J., Wu Y., Buffa J.A., Agatisa-Boyle B., Li X.S., Levison B.S., Hazen S.L. (2015). Gut Microbiota-Dependent Trimethylamine N-Oxide (TMAO) Pathway Contributes to Both Development of Renal Insufficiency and Mortality Risk in Chronic Kidney Disease. Circ. Res..

[37] Jameson E., Quareshy M., Chen Y. (2018). Methodological considerations for the identification of choline and carnitine-degrading bacteria in the gut. Methods.

[38] Rath S., Rud T., Pieper D.H., Vital M. (2020). Potential TMA-Producing Bacteria Are Ubiquitously Found in Mammalia. Front. Microbiol..

[39] O’Callaghan A., van Sinderen D. (2016). Bifidobacteria and Their Role as Members of the Human Gut Microbiota. Front. Microbiol..

[40] Makki K., Deehan E.C., Walter J., Bäckhed F. (2018). The Impact of Dietary Fiber on Gut Microbiota in Host Health and Disease. Cell Host Microbe.

[41] Santacruz A., Collado M.C., García-Valdés L., Segura M.T., Martín-Lagos J.A., Anjos T., Martí-Romero M., Lopez R.M., Florido J., Campoy C. (2010). Gut microbiota composition is associated with body weight, weight gain and biochemical parameters in pregnant women. Br. J. Nutr..

[42] Xu M., Wang B., Fu Y., Chen Y., Yang F., Lu H., Chen Y., Xu J., Li L. (2012). Changes of Fecal Bifidobacterium Species in Adult Patients with Hepatitis B Virus-Induced Chronic Liver Disease. Microb. Ecol..

[43] Wu X., Ma C., Han L., Nawaz M., Gao F., Zhang X., Yu P., Zhao C., Li L., Zhou A. (2010). Molecular Characterisation of the Faecal Microbiota in Patients with Type II Diabetes. Curr. Microbiol..

[44] Murri M., Leiva I., Gomez-Zumaquero J.M., Tinahones F.J., Cardona F., Soriguer F., Queipo-Ortuño M.I. (2013). Gut microbiota in children with type 1 diabetes differs from that in healthy children: A case-control study. BMC Med..

[45] Peng Q., Huo D., Ma C., Jiang S., Wang L., Zhang J. (2018). Monosodium glutamate induces limited modulation in gut microbiota. J. Funct. Foods.

[46] Mayengbam S., Chleilat F., Reimer R.A. (2020). Dietary Vitamin B6 Deficiency Impairs Gut Microbiota and Host and Microbial Metabolites in Rats. Biomedicines.

