# Supplementary material for: Monosodium Glutamate Induces Changes in Hepatic and Renal Metabolic Profiles and Gut Microbiome of Wistar Rats
Source: Nutrients. 2021 May 30;13(6):1865. doi: 10.3390/nu13061865 (PMC8229789; doi:10.3390/nu13061865)
Supplement: Supplementary file 1 [file nutrients-13-01865-s001.zip › nutrients-1195434-supplementary.pdf]

# Supplementary file

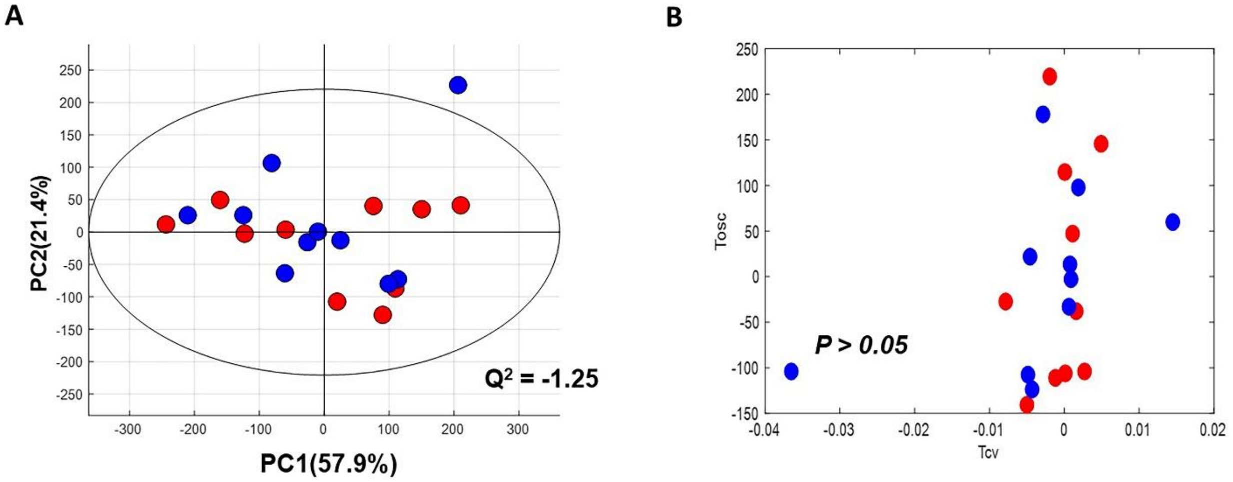


**Figure S1.** Principal component analysis (A) and O-PLS-DA score plot (B) of faeces of MSG treated (Red) and control groups (Blue). % PC represents variation explained by each principal component and $Q^2$ represents predictive ability of the model.

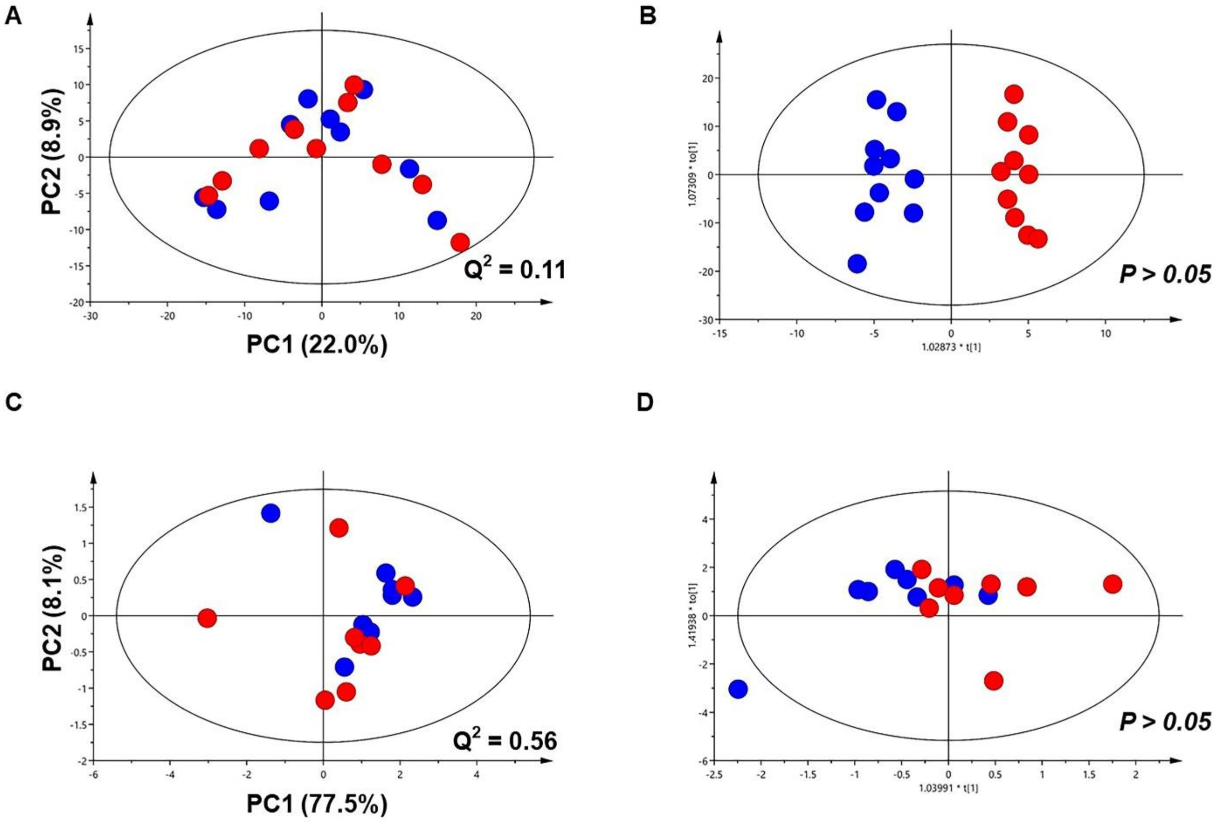


**Figure S2.** Principal component analysis (left panel) and O-PLS-DA score plot (right panel) of plasma after MSG treatment (Red) compared to control group (Blue). (A-B) positive ionization mode and (C-D) negative ionization mode. % PC represents variation explained by each principal component and $Q^2$ represents predictive ability of the model.

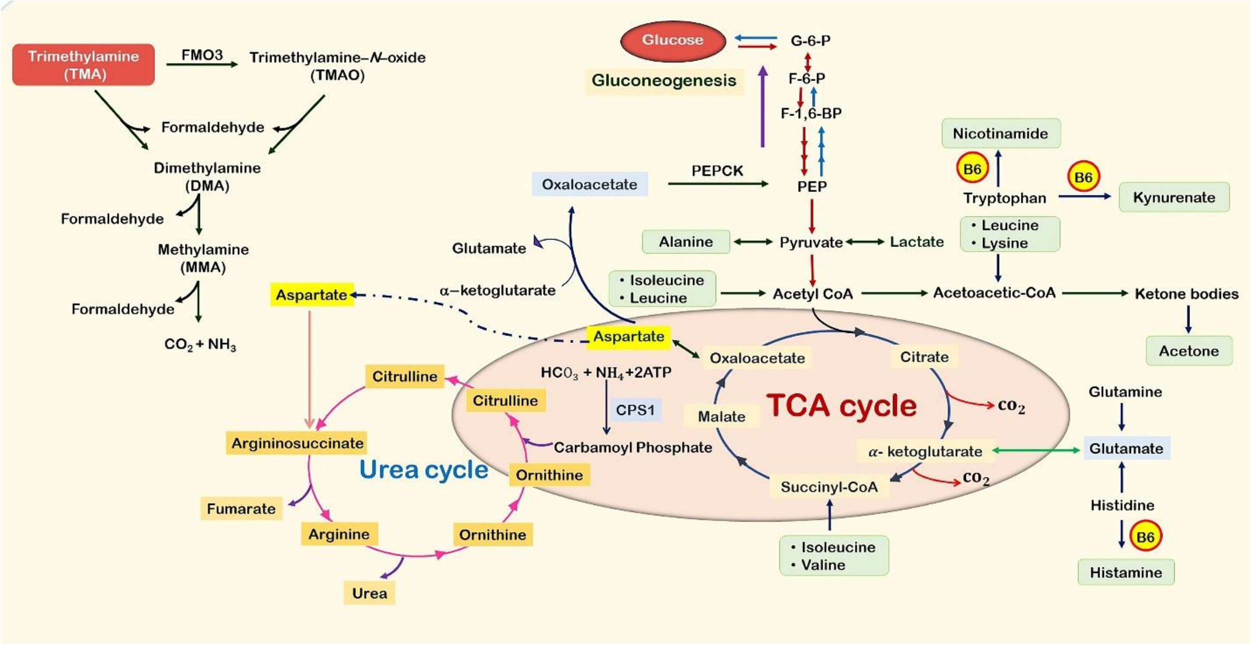


**Figure S3.** Pathway analysis of significant metabolites in rat tissues after MSG-treatment. Red frames indicate increased metabolites and green frames indicate decreased metabolites. Abbreviations: G-6-P, glucose-6-phosphate; F-6-P, fructose-6-phosphate; F-1,6-BP, fructose-1,6-bisphosphate; PEP, phosphoenolpyruvate; PEPCK, phosphoenolpyruvate carboxykinase; CPS1, carbamoyl phosphate synthetase1; FMO3, Flavin-containing monooxygenase.
